# Terminology - glossary including acronyms and quotations in use for the conservative spinal deformities treatment: 8^th ^SOSORT consensus paper

**DOI:** 10.1186/1748-7161-5-23

**Published:** 2010-11-02

**Authors:** Theodoros B Grivas, Jean Claude de Mauroy, Stefano Négrini, Tomasz Kotwicki, Fabio Zaina, James H Wynne, Ian A Stokes, Patrick Knott, Paolo Pizzetti, Manuel Rigo, Monica Villagrasa, Hans Rudolf Weiss, Toru Maruyama

**Affiliations:** 1 Orthopaedic and Trauma Department, "Tzanio" General Hospital of Piraeus, Zanni and Afendouli 1, Piraeus, Greece; 2 Clinique du Parc, Lyon, France; 3 ISICO (Italian Scientific Spine Institute), Milan, Italy; 4 Department of Paediatric Orthopaedics and Traumatology University of Medical Sciences, Poznan, Poland; 5 Boston Brace/National Orthotic Prosthetic Company, USA; 6 University of Vermont, Department of Orthopaedics and Rehabiliation, Burlington, VT 05405-0084, USA; 7 Rosalind Franklin University of Medicine and Science, Chicago, USA; 8 ISICO (Italian Scientific Spine Institute), Milan, Italy; 9 Institut E. Salvá, Barcelona, Spain; 10 Orthopedic Rehabilitation Services, D-55457 Gensingen, Alzeyerstr. 23, Germany; 11 Department of Orthopaedic Surgery, Saitama Medical Center, Saitama Medical University, Saitama, Japan

## Abstract

**Background:**

This report is the SOSORT Consensus Paper on Terminology for use in the treatment of conservative spinal deformities. Figures are provided and relevant literature is cited where appropriate.

**Methods:**

The Delphi method was used to reach a preliminary consensus before the meeting, where the terms that still needed further clarification were discussed.

**Results:**

A final agreement was found for all the terms, which now constitute the base of this glossary. New terms will be added after being discussed and accepted.

**Discussion:**

When only one set of terms is used for communication in a place or among a group of people, then everyone can clearly and efficiently communicate. This principle applies for any professional group. Until now, no common set of terms was available in the field of the conservative treatment of scoliosis and spinal deformities. This glossary gives a common base language to draw from to discuss data, findings and treatment.

## Background

The glossary used for communication within every professional group is as important as the language used for communication by the people of a country and culture of a group of people within a country. If different languages are used in a discussion, then the outcome is a "Biblical Tower of Babylon" where everyone miscommunicates. In contrast, when only one language is used for communication in a place or among a group of people, then everyone can clearly and efficiently communicates. The same principle applies for any professional group, and the common language included a specific set of terminology that is standard within that profession.

In the field of idiopathic scoliosis, after the first pioneering effort in Spine 1976 [1], the Scoliosis Research Society (SRS) has provided a great deal of work, with the SRS Revised Glossary of Terms [2], the SRS Biomechanichs Glossary [3], and the SRS 3-D Terminology based on the Stokes 1994 paper [4], as well as a glossary for patients [5]. In this respect also the National Scoliosis Foundation, the US association for scoliosis patients, gave its contribution with its own Glossary [6]. Nevertheless, a careful examination provided by the authors of this paper allowed to conclude that a lot of the terms used in conservative scoliosis treatment are not included in these glossaries, and that there was the need to start a new effort in this respect. In fact, among professionals who treat scoliosis with conservative methods, there has been much miscommunication in the past. This report, with a collection of the most frequently used terms in the field, attempts to correct this and improve the effectiveness and efficiency of communication among these professionals.

The aim of this paper is to report on the "Terminology in use for the conservative spinal deformities treatment" [7]. This is intended as an ongoing effort, to be pursued in time by the SOSORT and its Journal "Scoliosis" during its scientific and educational activities.

## Method

The Delphi method was used because it is a systematic, interactive forecasting method which relies on a panel of experts as described by Jones and Hunter [8]. The list of terms was prepared and distributed via e-mail among the SOSORT Board members and some other expert colleagues. The classification of the terms was organized into terms used for: Aim of treatment, Diagnosis, Evaluation, Brace Rehab and others.

The experts contributed with terms and definitions in several rounds. After each round, the dictionary was properly enriched and improved.

The best format for presenting each definition is to include text, followed by an illustration, picture, or figure and then a reference to the scientific literature.

An effort was make to apply this format to as many terms as possible. Pertinent terms from other existing related society dictionaries were also used and cited [1,2,5,6,9-13].

The illustrations/pictures/figures used here were modified from the original and the sources from where they were taken are cited.

When there were more than one accepted definitions for a single term, then they were all used when possible.

Before discussing this consensus topic, a rather deep discussion occurred during the SOSORT 2010 Board meeting on the issue. Then a print out with each term and definition was distributed to all SOSORT meeting participants in Montreal, and discussed during the Consensus Session on May 20^th^, 2010. Questions on terms were raised and discussed. Alternatively they could give some written suggestions for terms or definitions they felt should be added. These were collected and processed for the final manuscript.

## Results

Following this Delphi method, we are now able to discuss scoliosis treatment using a common set of terms and definitions, [Additional File 1]. These terms now constitute the base of an online glossary which is posted on the SOSORT website, and which will frequently be updated after receiving suggestions from experts in the field. Every suggestion will be discussed by a SOSORT assigned committee and after a consensus is reached the new term will be published. Wherever possible, pictures and illustrations are used to make the terms more understandable. The number of pictures will also be increased over time.

## Discussion

When only one language is used for communication in a place or among a group of people, then everyone can clearly and efficiently communicate. This same principle applies for any professional group. Until now, even in front of quite a large effort on scoliosis terminology [1-5,9-14] no common language was available in the field of conservative treatment of scoliosis and spinal deformities. This glossary gives a common base and a common language, and its use will facilitate discussion regarding research, data, findings and treatments.

The Delphi Method, already used for other SOSORT Consensus Papers [15-22] was a good way to discuss and reach a consensus before the meeting, and resulted in an efficient pathway to a common position. Some terms, in fact, could be interpreted in slightly different ways according to the background of each expert. Now the experts can speak a common language and this may lead to a growth in this field. The authors hope that this brings about a better understanding of what is practically done by other colleagues.

## Authors' contributions

TBG, SN and the SOSORT Clinical Committee were involved in the initial creation of this dictionary. TBG worked in the successive improvement of this dictionary, chaired this consensus paper at the Montreal, Canada SOSORT Meeting, May 19 - 22, 2010, involved in literature collection and contributed in drafting the manuscript. JCdeM was involved in the initial creation and successive improvement of this dictionary, processed the collected data on a web form, involved in literature collection and contributed in drafting the manuscript. All co-authors contributed in some way to the improvement of the dictionary and in drafting the manuscript. The other members of the SOSORT board contributed by reviewing, text editing and adding certain text files and references. All authors have read and approved the final manuscript.

## Supplementary Material

Additional file 1 **A Glossary of terms and definitions for the conservative treatment of spinal deformities**. This additional file is a common set of terms and definitions, which constitute the base of a glossary used for the conservative treatment of spinal deformities. Pictures and illustrations are used to make the terms more understandable. Click here for file
