# Supplementary material for: Terminology - glossary including acronyms and quotations in use for the conservative spinal deformities treatment: 8th SOSORT consensus paper
Source: Scoliosis. 2010 Nov 2;5:23. doi: 10.1186/1748-7161-5-23 (PMC2984375; doi:10.1186/1748-7161-5-23)
Supplement: Additional file 1 — A Glossary of terms and definitions for the conservative treatment of spinal deformities. This additional file is a common set of terms and definitions, which constitute the base of a glossary used for the conservative treatment of spinal deformities. Pictures and illustrations are used to make the terms more understandable. [file 1748-7161-5-23-S1.PDF]

| A | B | C | D | E | F | G | H | I | J | K | L | M | N | O | P | Q | R | S | T | U | V |

| W | X | Y | Z | Acronym | Quotations | edit form modifications |

Term	Definition
A	
Abbott's Frame or Cotrel's Frame	Frame used in 1913 by *Edville Gerhardt Abbott* for the treatment of scoliosis by lateral pulling and counterpulling on the spinal column by means of wide bandages and pads, until the deformity is over-corrected, and then applying a plaster jacket to produce pressure, counterpressure, and fixation of the spine in its correct position.<br>The frame was perfected by Yves Cotrel using the three-dimensional principles of the elongation, derotation and flexion.<br>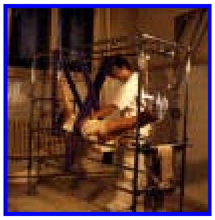 Abbott's frame 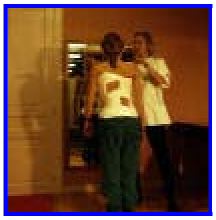 Plaster cast
Abdominal apron	Refers to the anterior portion of the brace that extends enough laterally and cephalad to contain the abdomen and just barely cover the margins of the ribs and xyphoid process.
Acceptability (brace)	One of the principles of bracing: a good brace should be as much acceptable as possible to increase compliance and consequently efficacy.
Active Self-Correction (ASC) (rehab)	The ability to reduce the spinal deformity through the postural realignment of the spine. It’s the core of the exercise treatment for scoliosis.<br>A movement actively and autonomously (without external aids such as sticks, wallbars or PT's hands) performed by the patient in order to achieve the best realignment of the spine in the three dimension.<br>The series of movements of realignment as a whole that the patients autonomously performs in order to reduce the scoliotic curves. These movements have to be performed as much as possible in three dimensions. ASC is the form of autocorrection proposed in SEAS exercises.<br>Technique of scoliosis correction - movement of correction used in physiotherapy of idiopathic scoliosis. Aim: making the vertebral column moving in the desired direction and maintenance in the corrected position. Actively performed by the patient. Consists of activation of muscles of the trunk and limbs resulting in movement of the vertebral column executed in the three planes of the space: in the coronal plane the lateral deviation is corrected, in the sagittal plane the physiological curvatures (thoracic kyphosis or lumbar lordosis) are promoted, in the transverse plane the axial plane deformity is being reduced. Autocorrection is considered by SOSORT experts the

	key technique in scoliosis physiotherapy (SOSORT Consensus).
Activities of Daily Living (ADL) (brace, rehab)	The things normally done in daily living including any daily activity we perform for self-care (such as feeding ourselves, bathing, dressing, grooming), work, homemaking, and leisure. 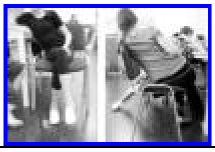
Adams Test (evaluation)	The Adam's Forward Bend Test is a standard exam that is often used by physicians and in initial school screenings. For this test, the patient is asked to lean forward with his or her feet together and bend 90 degrees at the waist. The examiner can then easily view from this angle any asymmetry of the trunk or any abnormal spinal curvatures.<br>The Adam's Forward Bend Test can be administered easily by school nurses or parent volunteers. It should be noted that this is a simple screening test that can detect potential problems, but cannot determine accurately the exact severity of the deformity. 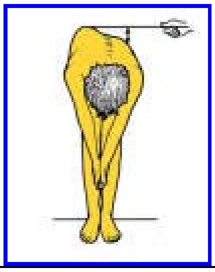
Adaptability (brace)	The possibility to use a brace for different pathologies and adapt it to different trunk conformations.<br>One of the principles of bracing: a good brace should be as much adaptable as possible to allow individual modifications as needed.
Adolescent Scoliosis	Lateral spinal curvature that appears before the onset of puberty and before skeletal maturity.<br>Threedimensional spinal curvature, that include rotation, lateral flexion and sagittal plane changes, that is discovered from the age of 10 years before the end of growth.
Adult Scoliosis (AS)	Scoliosis of any cause which is present after skeletal maturity.
Adult Onset Scoliosis	From age 18 and beyond
Aesthetics (aim)	The physical appearance of the trunk, and particularly of the back. It refers to the degree of asymmetry of the trunk.
Angle of Trunk Inclination (ATI)	with the trunk flexed to the horizontal, the angle between the horizontal and the plane across the back at the greatest elevation of a rib prominence or lumbar prominence, as measured by an inclinometer (scoliometer).<br>Synonimous of ATR.
Angular Balance	Is the lateral angulation of the most cephalad vertebra.
Antalgic Scoliosis	A spinal postural change that simulate a scoliosis in order to avoid a pain. The main features are the absence of the hump/prominence and the presence of pain.
Anterior	The front portion of the vertebral body. It may also indicate the position of one structure relative to another.<br>On the front side of the subject.
Anteroposterior View (AP View)	An x-ray in which the patient faces toward the x-ray beam, which passes from anterior to posterior through the patient, and away from the x-ray film.

Apex of Scoliosis	The area of greatest curvature or displacement from the midline of the body.
Apical Vertebra	In a curve, the vertebra most deviated laterally from the vertical axis that passes through the patient's sacrum, i.e. from the central sacral line.
Apical Disc	In a curve, the disc most deviated laterally from the vertical axis of the patient that passes through the sacrum, i.e. from the central sacral line.
Apophysis	A growth plate which is not apparent on x-rays until the bone is maturing, when it begins to ossify (change to bone). The iliac apophysis is often used to estimate a child's skeletal maturity.
Arrows (evaluation)	The difference of distance from the plumbline measured at C7, D12, L3 and S1 with respect to the apex of the thoracic kyphosis.
ATI – Angle of Trunk Inclination (evaluation)	With the trunk flexed to the horizontal, the angle between the horizontal and the plane across the back at the greatest elevation of a rib prominence or lumbar prominence, as measured by an inclinometer (scoliometer)
ATR – Angle of Trunk Rotation (evaluation)	The surface measure of the spinal rotation.
Autocorrection (rehab)	See also Active self correction<br>The main point of specific physical exercises for scoliosis. It can be reached in different ways according to the specific existing schools of treatment.
Autoelongation (rehab)	The attempt to reduce the spinal curvature by correcting the posture of the spine in the direction of the extension of the spine. It's a bi-dimensional correction of the scoliosis performed in the sagittal and frontal planes.
Awareness of the Deformation (rehab)	The consciousness of the deformity that is necessary to teach the patients the rehab treatment.<br>To teach Self perception of the curvature in terms of pathological knowledge (i.e. teaching the patient what scoliosis is with the aid of a model) and in terms of correspondence between what the patient see and what the patient feel and what the patient behave. The patient learns what is scoliosis, how it affects his/her body and how to deal with scoliosis at an emotional level.
Axillary extension	Refers to the portion of the completed brace intended to contact the lateral aspect of upper thoracic ribs from one vertebral level superior to thoracic null point cephalad. Generally there is an open 'window' between the axillary extension and the crest roll.
Axillary Plumb Line (evaluation)	*See balance*

B	
Back Surface Rotation (BSR)	
Balance	The word balance means different things to different people. From the point of view of the spine, it implies that, in both the frontal and sagittal planes, the head is positioned correctly over the sacrum and pelvis, in both a translational and angular sense. In the sagittal plane, the 'correct' balance is not necessarily zero, and it changes continuously as a result of

	postural sway (McGlashen et al., 1991). Posture is less reproducible in young children (Ashton-Miller et al. 1992). From the point of view of the trunk, balance implies that the shoulders are horizontal, and that the mass of the trunk is evenly distributed about the vertical line passing through the sacrum (the vertical global axis)<br>Thus "balance" implies a static alignment of a person in the standing (or unsupported seated) position. "Compensation" signifies the active process of becoming balanced, and "decompensation" signifies a failure to achieve balance, especially after an intervention such as surgery.<br>Balance does not exist at a local level. Usually, it is a property of the whole spine. However, at a regional level, a failure of both of the end vertebrae of a curve to lie on a global vertical axis could signify a regional lack of balance.<br>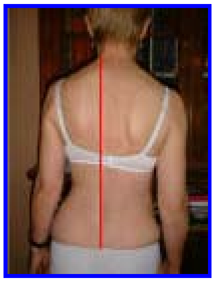 Frontal imbalance
Balance (offset)	Can be defined both as a distance and an angle. The displacement of the most cephalad vertebra from the global vertical axis (offset) can be measured as a distance from the global axis system or as an offset angle between the global and spinal axis systems.<br>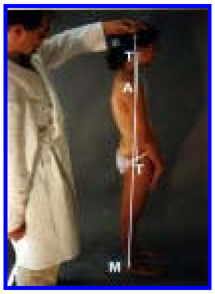 Sagittal balance
Bench Alignment	Static alignment of prosthetic/orthotic components.
Block Vertebra(e)	Block vertebrea occur when there is improper segmentation of the vertebrae, leading to parts of or the entire vertebrae being fused. It can lead to an angle in the spine, but there are usually no symptoms. The sacrum is a normal block vertebrae.<br>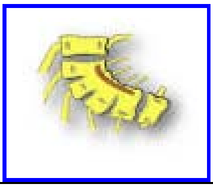
Body Cast	A cast which surrounds the chest, abdomen and pelvis. It may also include the shoulders. This may be used to correct scoliosis in very young patients or for postoperative spinal immobilization.<br>According to some schools, it can be used also in adolescents and adults.

Bone age (evaluation)	The degree of skeletal maturity. Usually this parameter is evaluated trough the Risser sign.<br>even if it is not accurate as the wrist evaluation according to the Greulich and Pyle Atlas.
Bone Spur	An overgrowth of bone in response to stress or injury.
Boston Brace	Symmetrical thoracolumbral sacral orthosis used in the conservative treatment of scoliosis. First introduced in 1977 by Watts, Hall, Stanish.
Brace	Another term used to describe a trunk **orthosis**.<br>An appliance that gives support to moveable parts.<br>*see also Dynamic Brace, Rigid Brace*
Breathing Function (rehab)	An aim of scoliosis rehabilitation, mainly in very important scoliosis in all ages, and during orthotic treatment.
Butterfly Vertebra(e)	Butterfly vertebrae have a cleft through the body of the vertebrae and a funnel shape at the ends. This gives the appearance of a butterfly on an x-ray. It is caused by persistence of the notochord (which usually only remains as the center of the intervertebral disc) during vertebrae formation.<br>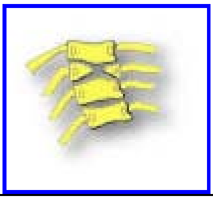

C	
C.T.O.	Cervico-Thoraco Orthosis.
C7 plumbline (evaluation)	The difference of the distance from the plumbline at C7 and at the apex of the thoracic kyphosis.
C7 shift (evaluation)	The difference of the distance from the plumbline at C7 and at S1 level in the frontal plane.
CAD CAM mill	Machine that carves to a specific shape.
CADCAM (COP)	Computer Aided Design, Computer Aided Manufacture.
Center of Mass (COM)	
Center of Pressure	
Cervico-thoracic Kyphosis (diagnosis)	A kyphosis involving the distal cervical vertebrae, causing and anterior protrusion of the head.
CervicoThoracoLumboSacral Orthosis (CTLSO)	A type of brace which immobilizes the cervical, thoracic and lumbar spine. This may be used to help stabilize/ prevent progression of scoliosis curve(s) while a child is growing, or to immobilize the spine after surgery.
Check (of a brace)	The test of the interaction of the brace and the trunk of the patient in order to improve the efficacy and the tolerance. During brace construction is the key moment in which the new brace is tested for the interaction with the trunk of the patient, in order to improve its efficacy and tolerance. It is up to the responsibility of the treating physician, and it is based on a strict collaboration between physician, orthotist, patient and family. It include counseling to allow proper compliance.
Chêneau Brace	Type of brace (external orthosis) for non-surgical management of progressive idiopathic scoliosis. Rigid brace constructed from polyethylene on individual measurements (custom built). Alternatively applies pressures over the trunk or creates free discharging spaces in specific areas of the body, following the

	rules introduced by Jaques Chêneau. Creates a system of 3-point pressure in order to achieve scoliosis correction in three dimensions. Used in combination with physiotherapy.<br>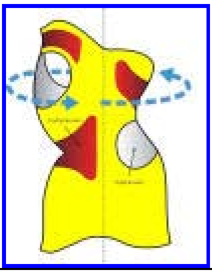
Chronologic Definition of Idiopathic Scoliosis presentations:	Infantile scoliosis - presenting from birth through age 2y+11m<br>Juvenile scoliosis - presenting from age 3 through age 9y+11m<br>Adolescent scoliosis - presenting from age 10 through age 17y+11m<br>Adult scoliosis - presenting from age 18y and beyond.
Cloth Gusset	Elastic cloth is affixed to an area of relief or window to provide a gradual transition between areas of pressure and relief, to provide limited pressure, or to maintain some anterior-posterior tension between the posterior and anterior parts of the brace, as between the abdominal apron and posterior uprights on a lumbar brace. Typical locations are above the iliac crest roll on both sides of a lumbar brace, or above the crest roll in the window below an axillary extension.
Cobb Angle (evaluation)	Cobb Method: angle between lines drawn on endplates of the end vertebrae (superior endplate of upper end vertebra; inferior endplate of lower end vertebra).<br>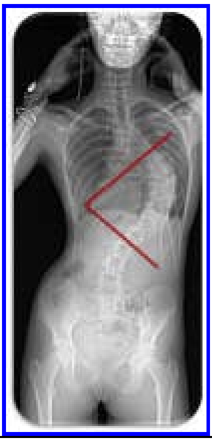
Cognitive-behavioural approach (rehab)	A therapeutic approach that aims at modifying the behaviour and psychological management of the patient with regards to his/her pathology.
Commitment to treatment (brace, rehab)	For the patient, the act of engaging himself for instance to wear the brace.<br>For the treating team, the strong belief in treatment needed to allow patients understand the importance of its treatment: a crucial element to achieve compliance, mainly in brace treatment.
Compensation	The vertical alignment of the mid point of C7 with the mid-point of the sacrum in the coronal plane (equates with the term coronal balance) i.e. horizontal distance of the C-7 mid point from the central sacral line.
Compensatory Curve	In spinal deformity, a secondary curve located above or below the structural curvature, which develops in order to maintain normal body alignment.

	It is defined in many different ways. A curve is secondary if it does not present rotation (hump), or if its value is at least 10° Cobb less than the primary one.
Competence (brace, rehab)	The experience in a specific medical area necessary for making diagnosis, prescribe and/or apply a treatment, follow up a patient.<br>The quality of being competent; adequacy; possession of required skill, knowledge, qualification, or capacity.
Compliance (brace, rehab)	The degree of concordance between the client's behavior and recommendations of health professions.<br>Apparently is a characteristic of the patient, in reality it depends on the behaviour of the treating team.
Compliancemeter (evaluation)	A manually operated mechanical device which can also be used as a pressure algometer.<br>An instrument to evaluate compliance: in bracing it can be inserted in the brace; in rehabilitation it can be a diary.
Concave (brace, rehab)	Curving inward or Curved like the inner surface of a sphere.<br>In scoliosis is usually considered the concave side of the curve looking at it on a bidimensional x-ray, but it must be recognised that the three-dimensional concavity is much more complex.
Congenital Scoliosis	Scoliosis due to bony abnormalities of the spine present at birth. These anomalies are classified as failure of vertebral formation and/or failure of segmentation.<br>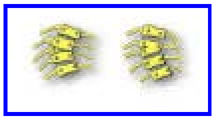 Segmental defect
Conservative Treatment	Is based on systematic medical monitoring and modification of management recommendations, depending on the needs. This type of treatment involves physiotherapy and bracing.
Construction (of a brace)	The act or result of interpreting the blueprint of a brace.<br>The process, art, or manner of making a brace.<br>Based on the indications of the physician, is the responsibility of the orthotist.
Contractures (rehab)	A contracture is the tightening of a muscle that deems it dysfunctional.<br>A condition of abnormal shortening or shrinkage of a muscle, tendon, etc., often with persistent flexion or distortion at a joint.
Convex (brace, rehab)	Having a surface or boundary that curves or bulges outward, as the exterior of a sphere.<br>In scoliosis is usually considered the convex side of the curve looking at it on a bidimensional x-ray, but it must be recognised that the three-dimensional convexity is much more complex.
Coordination (rehab)	Harmonious functioning of muscles or groups of muscles in the execution of movements<br>It depends on the neuro-motorial control
Coronal Plane (evaluation)	Reference plane as seen from the front, perpendicular in relation to sagittal and horizontal planes.
Coronal T1 Tilt Angle	The left/right tilt of the T1 vertebral body seen in the coronal plane on the AP radiograph. *(see also Sagittal T1 Tilt Angle)*

	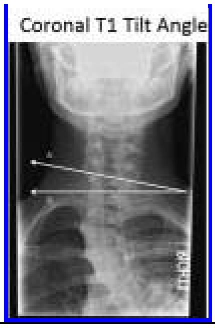
Coronal Vertical Axis (CVA)	The deviation of the plumbline from the odontoïd process in the coronal plane to the left or right of the center sacral line, measured in millimeters.<br>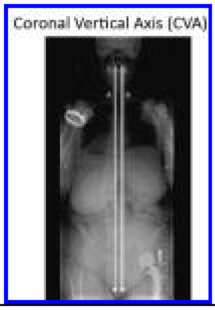
Corrected Posture (rehab)	The act of correct the posture to obtain the alignment of body parts supported by the right amount of muscle tension.<br>Proper manipulation of the joints to facilitate a sustainable, comfortable, health-promoting upright position.
Correction (of a brace)	In-brace correction: reduction of a vertebral deviation in a brace.
Corset	Lumbar brace made from textile material.
Counselling (brace, rehab)	The act of exchanging opinions and ideas.<br>Advice or guidance, especially as solicited from a knowledgeable person.<br>A process that Focuses on Helping You Understand Your Situation to Make Effective Decisions.<br>In spinal deformities during growth it is a crucial part of treatment, since usually they do not cause pain and motivation of the patient is not high. Counselling allows the patient and family to reach correct decision about their treatment, and consequently increase compliance both to bracing and rehabilitation (physiotherapy and exercises). It must be systematically made by all professionals involved in the treating team.
Counter-push (brace)	Push: part of the brace which apply pressure against the body for the purpose of correction. The counter-push is the part of the brace which apply a contrary pressure not opposite to the push, but complimentary to it and in the opposite side: combined with the push, allows correction.
Crude Trunk Asymetry Score (CTAS)	
Custom Fabricated Orthosis Custom Made	Orthosis, which is individually made for a specific patient. Created using an impression generally by means of plaster or fiber cast, a digital image using computer-aided design-computer aided manufacture (CAD-CAM) systems software, or direct form to patient.

D	
DAPI index (evaluation)	Deformity in the Axial Plane Index.
Decompensation	Compensation greater than a specified threshold value.<br>A failure to achieve balance, especially after an intervention such as surgery.
Deflexion (brace, rehab)	The property of being bent or deflected.<br>The bending which a beam or girder undergoes from its own weight or by reason of a load.<br>The action of straightening a scoliotic curve on the frontal plane.
Degenerative Scoliosis	Classification (Galssman 2005 - Schwab - 2005)<br>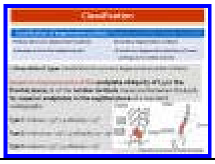
Dekyphotization (brace, rehab)	The action of reduction of the kyphosis of the spine.<br>Neologism. The act to correct an hyperkyphosis in a brace.
Delordosization (brace, rehab)	The action of reduction of the lordosis of the spine.<br>Neologism.<br>The act to correct an hyperlordosis in a brace.
De novo Scoliosis	A scoliosis appearing after bone age.
Derotation (brace, rehab)	The action of reduction of the vertebral rotation in a scoliotic curve.<br>The act to correct the scoliosis rotation manually or with a brace.<br>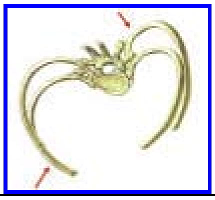
Disability (aim)	Disabilities is an umbrella term, covering impairments, activity limitations, and participation restrictions. An impairment is a problem in body function or structure; an activity limitation is a difficulty encountered by an individual in executing a task or action; while a participation restriction is a problem experienced by an individual in involvement in life situations. Thus disability is a complex phenomenon, reflecting an interaction between features of a person’s body and features of the society in which he or she lives.
Disc Wedge Angle	*See also Vertebral Wedge Angle*
Disharmonic (evaluation)	Without symmetry in physical body or attitude.<br>The reduction or loss of the ratio of two parameters.<br>Inside a physiologic curve (kyphosis and lordosis) it is a not harmonic distribution of the curvature among the involved vertebrae.
Distal	Situated away from or farther from a point of reference; opposite of proximal.<br>Term used to describe the furthest area from the body.
DoboMed physiotherapy	Method of physiotherapy for idiopathic scoliosis. Aim – performing active corrective movements of vertebral column. The key exercises comprise asymmetric, active, respiration guided movements of the rib cage, consisting of concave

	expansion followed by convex depression coupled with thoracic spine kyphotization. Used in combination with bracing or as exclusive treatment.
Double Scoliosis Curvature	Two lateral curvatures (scoliosis) in the same spine.
Double Major Scoliosis Curvature	Describes a scoliosis in which there are two structural curves which are usually of equal size.<br>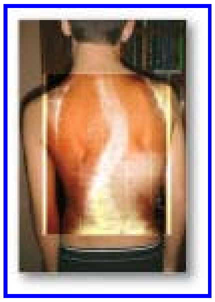 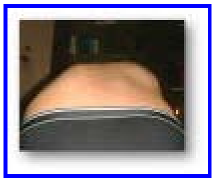 Double Major Scoliosis
Double Rib Contour Sign (evaluation)	(DRCS) All lateral standing spinal radiographs in idiopathic scoliosis show a DRC sign of the thoracic cage, a radiographic expression of the rib hump. The outline of the convex overlies the contour of the concave ribs.<br>The rib-index is the ratio d1/d2. d1 is the distance between the posterior margin of the vertebral body and the most extended point of the most projecting rib contour. d2 is the distance between the posterior margin of the same vertebral body and the most protruding point of the least projecting rib contour.<br>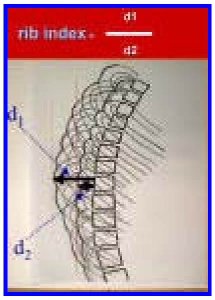 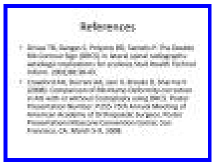 References [illegible]
Double Thoracic Scoliosis Curvature	A scoliosis with a structural upper thoracic curve, as well as a larger, more deforming lower thoracic curve and a relatively non-structural lumbar curve.
Dorsal	The back side of the trunk.
Drerup Rotation (evaluation)	Method to measure apical vertebral rotation (AVR) according to Drerup. (see also Nash & Moe, Perdriolle, Raimondi).
Driver (brace)	The portion of the brace without pushes whose aim is to drive the corrective force in the desired direction. It has been introduced by Stefano Negrini with the SPoRT concept of correction of scoliosis.
Duval-Beaupère' Linear Progression of Scoliosis	In 1979, Duval-Beaupere describes the linear evolution in 3 steps of the poliomyelitic scoliosis during the pubertal growth.<br>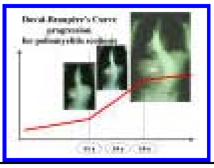
Duval-Beaupère' Pelvic Parameters	A Sacral slope angle (SS):angle between the upper plate of S1 and the horizontal line B<br><br>B pelvic tilt angle (PT):angle between the vertical line and the

	line connecting the midpoint of the upper plate of S1 to the femoral heads C<br><br>C pelvic incidence angle (PI): the angle between the line perpendicular to the sacral plate at its midpoint and the line connecting this point to the middle of the femoral heads.<br><br>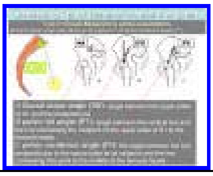
Dynamic Derotation Brace	The dynamic derotation brace (DDB) was designed in Greece in 1982, as a modification of the Boston brace. It is a custom-made, underarm spinal orthosis featuring aluminium blades set to produce derotating and anti-rotating effects on the thorax and trunk of patients with scoliosis.<br><br>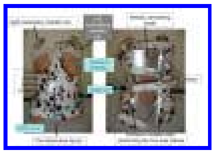 original paper

E	
Education of patient and family (rehab)	Act or process of imparting or acquiring general knowledge, to develop the power of reasoning and judgment.
Equality of Weight Bearing (rehab)	Ability of a part of the body to resist or support weight with a symmetric load distribution to the lower limbs.
Equilibrium (rehab)	A stable condition in which forces cancel one another. The therapeutical aim is to address the balance through enhancement of postural stability and motor control and self-perception.
End Vertebra	Concept: The cephalad and caudal vertebrae that bound a scoliosis curve, as seen in the frontal projection.<br>Definition: Cephalad end vertebra: The first vertebra in the cephalad direction from a curve apex whose superior surface is angled maximally toward the concavity of the curve, as measured in the PA spinal projection. Caudad end vertebra: the first vertebra in the caudad direction from a curve apex whose inferior surface is angled maximally toward the concavity of the curve, as measured in the PA spinal projection.
Ergonomy (rehab)	ADL based rehabilitation in respect of scoliotic patients specific needs. The therapeutical aim is to teach the patient how to adapt daily activities such as sitting or studying or wearing a backpack in respect of his spinal deformity. Can be considered also as a branch of cognitive behavioural therapy. Can address indirectly postural stability and motor control.
Escape (brace)	A direction toward which the thorax and the spine can move in order to recover the spinal alignment.<br>In a brace can be a window or a part of the brace adequately detached from the body so to allow the achievement of correction.
Exercises (rehab)	Exercise is physical activity that is planned, structured, and repetitive for the purpose of conditioning any part of the body.
Experience (brace, rehab)	Direct personal participation or observation; actual knowledge or

	contact.<br>The totality of a person's perceptions, feelings, and memories.
Extrinsic Autocorrection (rehab)	Exercise of correction used within many methods of physiotherapy in the treatment of spinal deformities. The self correction is induced with the help of tactile stimulation by the therapist or other kinds of aids or interventions. Extrinsic Autocorrection is used within the Schroth treatment as well as in the Scoliologic(TM) Best Practice programs [phase I], in order to enable the patients to exercise without any external help later on (see Intrinsic Autocorrection) and to gain the postural feeling necessary for keeping the corrections during the activities of daily living (see ADL) [phase II].

# F	
Fergusson Angle (evaluation)	Angle formed by the lines connecting the center of the apical vertebral body to the center of the end vertebrae of the scoliotic curvature.<br>It has an historical value, since today the Cobb angle has been adopted as the 'gold standard'.<br>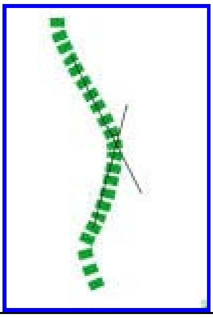
Flatback Syndrome/Fixed Sagittal Imbalance Syndrome	Forward posture usually due to a flattened lumbar spine from postoperative or degenerative changes. When viewed from the side, the patient's head may be several centimeters in front of their hips.
Follow-up	Regular clinical examination of a child, usually every 3-4 months in periods of growth spurt and every 6-12 months in other periods.
Full-time bracing (B 100%)	Wearing a brace all the time (at school, at home, in bed, etc.). From 20 to 24 hours per day.
Functional Scoliosis	A structurally normal spine that appears to have a lateral curve (scoliosis).<br>Nonstructural scoliosis involves a temporary change of spinal curvature. This is caused by an underlying condition such as a difference in leg length, muscle spasms, or inflammatory conditions, (e.g. appendicitis), which may produce muscle spasm. Functional scoliosis is treated by correcting the underlying problem. The spine itself needs no treatment. Functional scoliosis is also called nonstructural scoliosis as opposed to structural scoliosis in which there is a fixed curve of the bones of the spine (the vertebrae).<br>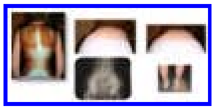 Leg length Discrepancy

G	
General Motor Capacity (rehab)	Term used to describe the global motor skills.
Gibbus	A localized kyphosis<br>Synonimous of hump.<br>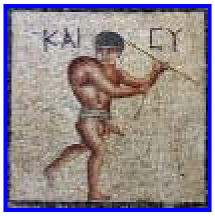 Antakaya Museum

H	
Harmonic (evaluation)	Term used to describe curves of kyphosis and lordosis balanced in the sagittal plane.<br>Moreover, inside a physiologic curve (kyphosis and lordosis) it is a proportional distribution of the curvature among the involved vertebrae.
Hemivertebra	A congenital abnormality of a vertebral body caused by incomplete development of one side of a vertebra. Usually a wedge shape which causes scoliosis or kyphosis.<br>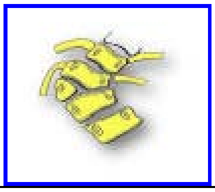
Hollow Back (diagnosis)	Non technical name for Lordosis: an abnormal inward (forward) curvature of the vertebral column.
Hump (evaluation)	The asymmetry of the height of one side of the spine with respect to the other during the forward bending test. It' caused by the rotation of the vertebrae and the ribs.<br>It's height can be measured in millimeters (same distance on the two sides of the spine, with the level at the maximum height of the hump), while the inclination among the two sides can be measured with inclinometers (ATI or ATR).
Hump Sum (evaluation)	Surface topography parameter.
Hyperkyphosis	Refers to an abnormal increase in this forward curvature.
Hyperlordosis (diagnosis)	A lordosis greater than the normal range.
Hypertonicity (rehab)	Increased muscle tone or muscle tension.
Hypokyphosis	A kyphosis of the thoracic spine less than the normal range.
Hypolordosis (diagnosis)	A lordosis of the cervical or lumbar spine less than the normal range.
Hypotonicity (rehab)	Loss of muscle tone (or tension).
Hysterical Scoliosis	A non-structural deformity of the spine that develops as a manifestation of a psychological disorder.

	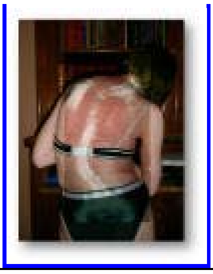

I	
Iliac Crest Roll	The inward protruding roll of plastic and padding which comes to rest midway between the iliac crest and the lower margin of the ribs. Its function is to prevent distal or proximal migration of the brace, and to aid in positioning the pelvis in a posterior directed tilt.
Idiopathic Scoliosis	A structural spinal curvature for which the cause has not been established. There is no evidence of underlying physical or radiographic pathology. The most common type of scoliosis.
Imbalance	
Improvement (brace, rehab)	The act or process of improving.<br>The state of being improved.<br>It's one outcome of scoliosis treatment that can be achieved, although it is usually proposed only stability.
In-brace X-ray (evaluation)	A radiographic picture taken while wearing an orthosis. It is taken to evaluate the effects of the orthosis body segment.<br>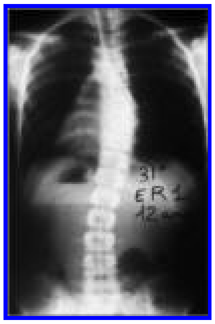 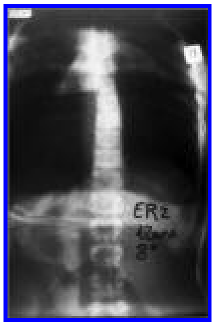 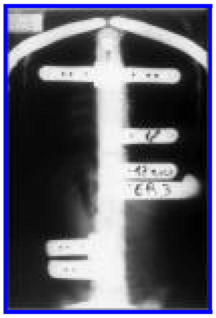 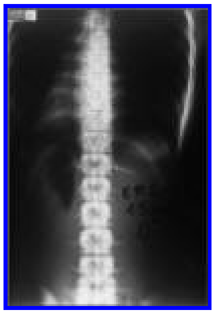
Inclinometer	An instrument used to measure the angle of thoracic (rib) or lumbar (flank) prominence, referred to as the angle of trunk rotation (ATR). (also known as a scoliometer)
Infantile Scoliosis	A curvature of the spine that develops before three years of age.
Interspinal or Intervertebral disc	The structure that normally occupies the space between two moving vertebrae. It is more prominent in the cervical and lumbar spines. It is much like a radial tire. The centermost portion of the disc (nucleus pulposus) is normally composed of a clear gelatinous material that varies in consistency from a firm jelly material to a very thick and less pliable substance. This core is then surrounded by numerous layers of fibrous (fibrocartilaginous) material called the annulus fibrosus. That structure goes to the normal margins of the vertebral body. There is a thick ligament (approximately 2mm) that covers the anterior part of the vertebral body called the anterior longitudinal ligament, and on the spinal canal side posteriorly is the posterior longitudinal ligament.
Intrinsic Autocorrection (rehab)	Corrective exercises / movements performed without external help. Extrinsic Autocorrection is used within the Schroth

	treatment as well as in the Scoliologic(TM) Best Practice programs first [phase I], in order to enable the patients to exercise without any external help later on and to gain the postural feeling necessary for keeping the corrections during the activities of daily living (see ADL) without external help [phase II]. 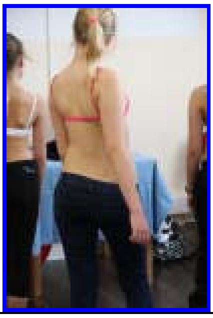
I.S.I.S. ®	Integrated Shape Imagine System. 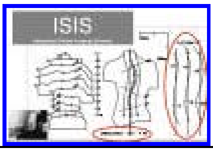

J	
Juvenile Scoliosis	Scoliosis diagnosed between 3 years and 9 years and 11 months of age.

K	
Kyphoscoliosis	A structural scoliosis associated with increased kyphosis (roundback).
Kyphosis	Posterior convex curvature of the spine. Rounded hump on top of back.<br>Kyphosis and Lordosis are descriptions of shape only. For abnormal (undesirable) curvatures, use hyperkyphosis and hypokyphosis.<br>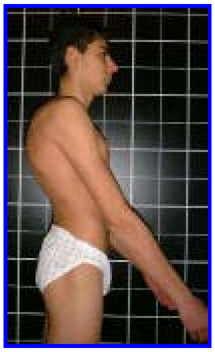 Hyperkyphosis
Kyphotization (brace, exercises)	An attempt to recover the thoracic kyphosis that is usually reduced in the thoracic scoliosis.

	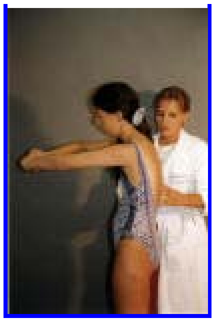 Kyphotization exercise

L	
L.S.O.	Brace used to support or immobilize the lower back (Lumbo-Sacral-Orthosis).
Lateral	Situated away from the midline of the body.<br>Toward the outside, away from center.
Leg length Discrepancy (evaluation)	When one leg is longer than the other.
Lehnert-Schroth Augmented Classification	A simple classification derived from the original in order to allow a pattern specific brace construction.<br>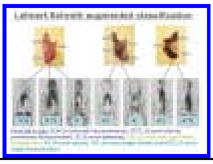
Longitudinal	In lengthwise direction.
Lordoscoliosis	A lateral curvature of the spine associated with increased lordosis (swayback).
Lordosis	Anterior convex curvature of the spine.<br>The normal mild anterior angulation (swayback) of the lumbar spine as evaluated from the side. Contrast to kyphosis. Kyphosis and Lordosis are descriptions of shape only. For abnormal (undesirable) curvatures, use hyperlordosis and hypolordosis.<br>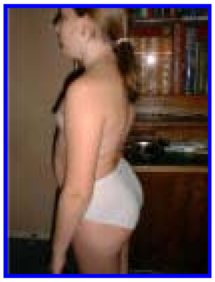 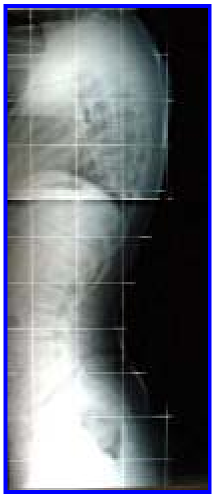
Lordotization (brace, exercises)	An attempt to recover the lumbar lordosis that is usually reduced in the lumbar scoliosis
Lower back	Lumbo-sacral area of the back.
Lumbar Scolisis Curve	A spinal curvature whose apex is between the first and fourth

	lumbar vertebrae (also known as lumbar scoliosis).<br>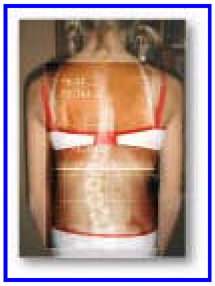 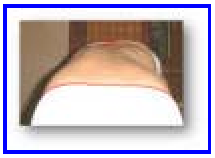 Lumbar Scoliosis
Lumbar Spine	Five mobile segments of the lower back (L1 to L5). These are the largest of the vertebral segments and provide most of the bending and turning ability of the back, in addition to bearing most of the weight of the body.
Lumbosacral	Pertaining to the lumbar and sacral regions of the back
Lumbo-Sacral Angle (evaluation)	Angle formed between the plane of the superior surface of S1 to the horizontal plane.<br>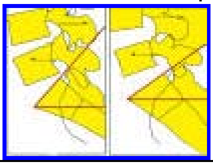
Lumbosacral Scoliosis Curve	A lateral curvature with its apex at the fifth lumbar vertebra or below (also known as lumbosacral scoliosis).
Lyon Brace	The Lyon brace is used since 1947 for adolescent scoliosis. It is adapted after realization of a plaster cast.<br>The brace is adjustable, symmetrical, stable, transparent.<br>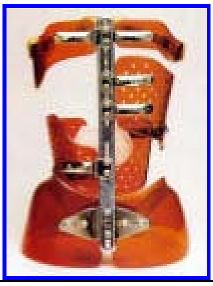

M	
Manipulation (rehab)	The action of touching with the hands or the skillful use of the hands.<br>The osteopathic spinal manipulations have been described by Andrew Taylor Still.
Major Scoliosis Curvature	The curve with the largest Cobb measurement on upright long cassette coronal x-ray of the spine.
Medial	Situated closer to the midline of the body.<br>Towards the center line, middle.
Mehta Rib Vertebra Angle (evaluation)	The rib vertebral angle (RVA) measurement was first introduced by Mehta in 1972 as a means of determining spinal rotation in a scoliotic curve for infantile scoliosis. The method involves careful measurement of the rib-vertebra angles at the apex of the curve and observation of the relationship of the head of the rib to the body of the vertebra as seen in antero-posterior radiographs.

	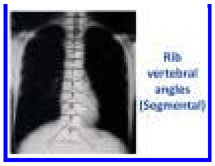 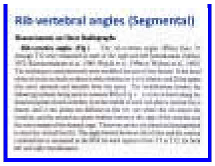 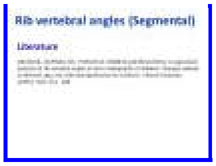
Melatonine	
Milwaukee Brace	The Milwaukee brace, also known as a cervico-thoraco-lumbo-sacral orthosis or CTLSO, is a back brace used in the treatment of spinal curvatures (such as scoliosis or kyphosis) in children. It is a full-torso brace that extends from the pelvis to the base of the skull. It was originally designed by Blount and Schmidt in 1946 for postoperative care when surgery required long periods of immobilization.<br>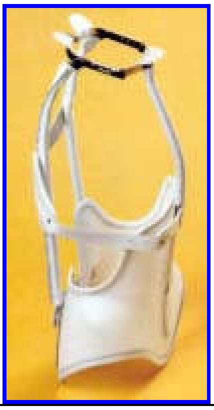
Minor Scoliosis Curve	Any curve that does not have the largest Cobb measurement on upright long cassette coronal x-ray of the spine.
Mobilization (rehab)	These are used by physiotherapists to treat joints that have become a little stiff from lack of movement, and are causing pain, such as low back pain resulting from stiffness in the lumbar spine. They are gentle, controlled movements, not manipulation, of the joints affected, with the aim to increase movement and relieve pain.
Modeling Push (brace)	Part of the brace that is sculpturing or forming the trunk in a pliable material, such as polyethylene or plexi.
Module	Refers to the prefabricated, symmetric, Boston Scoliosis Module. There are now a number of other thermoplastic prefabricated units available for rapid fabrication of Milwaukee braces and low profile, “underarm”, or TLSO braces. These units differ in shape, materials and design from the Boston Brace Module System. (Most published results and this manual refer to Boston Braces utilizing Boston Brace prefabricated modules. If other prefabricated systems are utilized, they should not be called Boston Braces. Only in this way can we avoid confusion and continue to evaluate the relative merits of different systems.
Multiprofessional Team (brace, rehab)	Multi-professional working that requires people from different professions to work together towards meeting (other profession than medical).
Multispecialty Team (brace, rehab)	A group organized to work together providing service in or staffed by members of several medical specialties (only medical profession).
Muscular Endurance (rehab)	The ability of a muscle or group of muscles to sustain repeated contractions against a resistance for an extended period.<br>Ability to make repeated contractions against a moderate load.
Muscular Strength (rehab)	The ability of a muscle or group of muscles to generate force in a short period of time.

	The force that is exerted by a muscle or a muscle group in a single contraction.

# N	
Nash and Moe Rotation (evaluation)	Analogic Scale which allows to measure vertebral rotation on a frontal radiograph using the displacement of the pedicle on the vertebral body. (see also Perdriolle, Drerup, Raimondi)<br>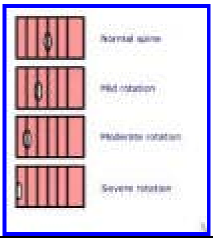
Negative Plaster Cast	Wrap cast, plaster or similar impression.
Nerve Root	The portion of a spinal nerve in close proximity to its origin from the spinal cord.
Neuromotorial Control of the spine (rehab)	Automatic control of posture.<br><br>The French term Neuromotorial is an innate response, controlled in space and time in which muscle contractions are organized in a comprehensive and automated system, allowing the body to adjust its position to an external stimulus (induced motor) or a voluntary change in position).
Neuromuscular Scoliosis	Scoliosis in children with any disorder of the neurological system. Common categories include cerebral palsy, spina bifida, muscular dystrophies, spinal cord injuries and so forth. Most of these children have as a unifying feature weakness of the trunk. As they grow and their trunk gets weaker, there is a progressive, collapsing deformity of the spine producing a long, c-type curve. These curves tend to be progressive, with the rate of progression becoming worse during rapid growth.<br>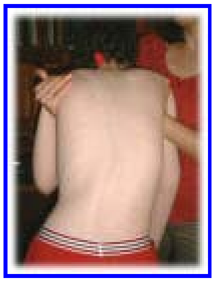 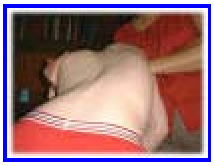 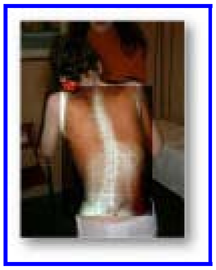 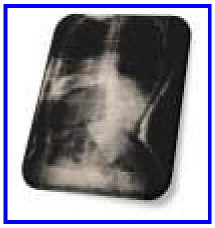
Night Brace<br>Night-Time Bracing (B 30%)	Orthosis used at night to keep specific body part in a certain desired position.
Nurse (others)	Is a healthcare professional, who along with other health care professionals, is responsible for the treatment, safety, and recovery of acutely or chronically ill or injured people, health maintenance of the healthy, and treatment of life-threatening emergencies in a wide range of health care settings.

# O	

Observation (rehab)	The act of noting and recording for instance the evolution of a scoliosis without treatment.<br>The first step of any medical evaluation, in the record of all the asymmetries and dermal signs of the body of the patients, principally focused on the trunk.<br>First step of scoliosis treatment, applied when a curve is not yet considered worthwhile a treatment, but still can evolve and need surveillance in time.
Orthopaedic Treatment (brace)	Treatment that deals with the prevention or correction of injuries or disorders of the skeletal system and associated muscles, joints, and ligaments. It includes the non-surgical conservative orthopaedic treatment and the orthopaedic surgery.<br>Classically used (in contrast to orthopaedic surgery) to indicate brace treatment.
Orthopaedic Surgery or Orthopaedics (also spelled orthopedics)	Is the branch of surgery concerned with conditions involving the musculoskeletal system.
Orthosis, (Orthoses)	Custom-fabricated or custom-fitted brace or support designed to align, correct, or prevent neuromuscular or musculoskeletal dysfunction, disease, injury, or deformity. Note: this does not include supports or devices carried in stock and sold by drug and other stores, corset shops or surgical supply facilities (e.g., fabric and elastic supports, corsets, arch supports, trusses, elastic hose, canes, crutches, cervical collars, dental appliances).
Orthotic (adj.)	The science and practice of evaluating, measuring, designing, fabricating, assembling, fitting, adjusting, or servicing an orthosis under a prescription from a licensed physician, chiropractor, or podiatrist to correct or alleviate neuromuscular or musculoskeletal dysfunction, disease, injury, or deformity.
Orthotics	The science and clinical service dealing with identifying clinical indication for orthotic treatment and the design, manufacture, fitting as well as clinical/technical maintenance as pertaining to orthoses.
Orthotist (others)	A person having gone through formal training and exam in orthotics.
Outpatient Clinic	Facility treating outpatients; clinic for patients not being admitted to stay.
Outpatient Physiotherapy	Special kinesitherapeutic methods and complementary physical therapy procedures carried out on an outpatient basis. (at a rehabilitation division, physiotherapy facility) or a similar form of health care (e.g. physiotherapy at home).
Out-of-brace X-ray (evaluation)	A radiograph taken in the absence of wearing an orthosis.
Overall Balance Summation (OBS)	

# P	
P.T.	Physical Therapist.
Pad	A pad is used to cushion or take up space in an orthosis. creating a cushion (upholstery, shock absorbing surface).
Paravertebral Height Difference	With the trunk flexed to the horizontal, the difference in height (in millimeters) between two points on the posterior rib cage. The two points are identified as (1) the most prominent point (2) a point on the opposite side of the back, at an equal

	distance from the midline.
Part-time Bracing (B 50%)	Wearing a brace outside school and in bed (approx. 12 hours daily).<br>When a brace is worn more than 12 hours per day (nighttime bracing) but less than 20 hours per day (full time bracing).
Peak Height Velocity (evaluation)	A point in pubescence in which the tempo of growth is the greatest.
Pelvic Incidence (evaluation)	Angle described by Duval Beaupere. It is formed by a line joining the centre of the sacral plate to the centre of the femoral head and the tangent to the sacral plate. It is the complementary angle of the pelvic tilt.<br>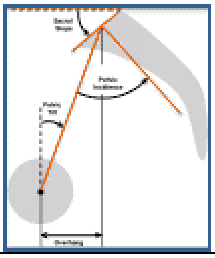
Pelvic Inclination (evaluation)	Deviation of the pelvic outlet from the vertical in the sagittal plane. Measured as an angle between the line from the top of the sacrum to the top of the pubis, and a horizontal line perpendicular to the lateral edge of the standing radiograph.
Pelvic Obliquity	Angulation of the pelvis from the horizontal in the frontal plane, possibly secondary to a contraction below the pelvis, e.g. of the hip joint. If this angulation is due to a leg length inequality, then the leg lengths should be equalized to create a level pelvis for measurement purposes.
Pelvic Tilt (evaluation)	One of the five major kinematic determinants of gait that lowers the pelvis on the side of the swinging lower limb during the walking cycle.<br>Pelvic tilt is defined as the angle between a vertical line and a line connecting the the centre of the femoral head to the centre of the vertebral plate. This angle is the complementary angle of pelvic incidence angle.<br>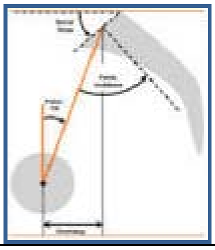
Perdriolle Rotation (evaluation)	One of the method to measure vertebral rotation with the Perdriolle torsiometer. (see also Nash & Moe, Drerup, Raimondi)<br>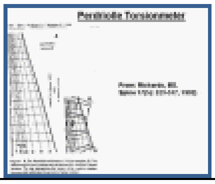
Photography (evaluation)	The process of producing images of a patient on photosensitive surfaces or digital device.
Physical and Rehabilitation Medicine (PRM), or Physiatry	Is a branch of medicine which aims to enhance and restore functional ability and quality of life to those with physical impairments or disabilities.
Physical Therapist (PT)	A trained professional who performs and teaches exercises and

	other physical activities to aid in rehabilitation and maximize physical ability with less pain. PTs teach the amputee exercise techniques, gait training and ways to navigate physical barriers with a prosthesis.
Physical Therapy (physiotherapy in many English speaking countries)	Health care profession which provides services to individuals and populations to develop, maintain and restore maximum movement and functional ability throughout life. 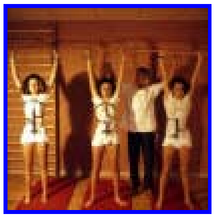 In brace physiotherapy
Plane of Maximal Curvature	
Plastazote	A material used for padding in O&P. Made up of microcellular polyethylene foam.
Plumb line	Vertical reference line. 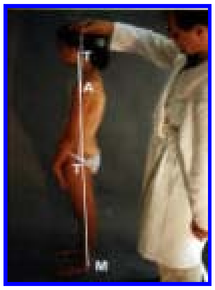 Alignment Tragus Acromion Trochanter malleoli
Polyethylene (PE)	A flexible type of plastic that is used in O&P.
Polypropylene (PP)	A more rigid type of plastic used in the fabrication of orthoses and prostheses.
Positive mold	A three-dimensional cast made of a plaster impression.
Posterior	Located behind a structure, such as relating to the back side of the body.<br>Behind, toward the back of the body.
Posterior Longitudinal Ligament	A ligament which attaches to the posterior aspect of every vertebra, from the base of the skull to the sacrum.
Posterior superior extensions ('rabbit ears')	*"Hypokyphosis Modification"* Refers to the module plastic and pad extending cephalad from the iliac crest roll and intended to contact lower thoracic ribs.
Postero-lateral	The back and outside of a specific object.
Postero-medial	The back and inside of a specific object.
Postural Self Control (rehab)	Ability to control one's posture, It can be increased through suitable exercise.
Posture (evaluation)	The position of the human body in the space. The relative positions of the human body parts one with respect to the other Relationship of various parts of the human body. Ideal posture requires less energy to be maintained and is advantageous for movements or static loading of the body.
POTSI index (evaluation)	Posterior Trunk Symmetry Index, parameter of assessment of the surface trunk deformity in scoliosis, described by Suzuki et al. (1, 2), is a key parameter to assess deformity in the coronal plane. Eight specific points at the surface of the patient's back are required. POTSI is relatively simple to measure, even on regular photography of the back. Ideal POTSI is zero, meaning

	full symmetry of the back surface. Normal values were reported to be below 27 (2,3). POTSI is very sensitive in revealing any frontal plane asymmetry.<br>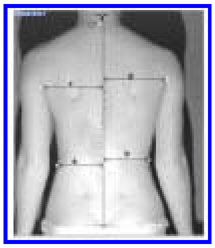 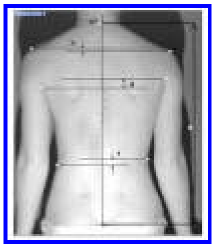 pdf
Pre-fabricated	Orthosis, which is manufactured in quantity without a specific patient in mind, which may be trimmed, bent, molded, or otherwise modified for use by a specific patient (i.e., custom fitted). A preformed orthosis is considered prefabricated even if it requires the attachment of straps and/or the addition of a lining and/or other finishing work or is assembled from prefabricated components is considered prefabricated. Any orthosis that does not meet the definition of a custom fabricated orthosis is considered prefabricated. Also referred to as custom-fitted.
Prescription (of a brace)	A written (by the treating physician) direction for the application of an orthosis and or physical therapy.<br>A written order by a doctor for a medical service or device.<br>If a concordance has not been already reached through a specific classification or other means, it requires on the side of physician clear specification on how and where the curve must be correct.
Primary Scoliosis Curve	Curve presenting with the largest cob angle and most rotation.
Procedure for Sitting Plane Radiographs:	Normally this must be done with AP projection. Similar precautions should be used to position the pelvis as the origin of the global axis system, and to support the arms out of the x-ray field.
Procedure for Standing Plane Radiographs	**PA projection** (for dose reasons), FFD = 2m (or 6 ft 6"), patient standing (if able to). The use of supports to position the ASIS parallel to the film plane is recommended to align the patient's global axis system with the film plane. X-ray central beam should be aimed at the 10th thoracic vertebra.<br>**Lateral projection**: Position patient as closely as possible to the same posture as was used for the PA projection. Arms should be supported in front of the patient<br>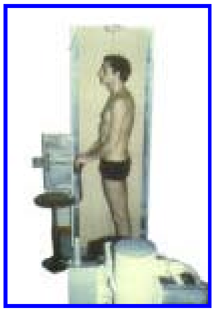 Original document<br>Film plane and central beam direction should be at 90° to that used for the PA projection.<br>Intermediate projections: e.g. plan d'élection (Stagnara 1984) as called for by specific measurement requirements.
Procedure for Supine Radiographs:	For patients unable to stand or sit (e.g. peri-operative films) appropriate precautions should be used to maintain the pelvis as the origin of axes.

Progression (brace, rehab)	Increasing in extent or severity.
Prominence (evaluation)	*See hump*
Proprioception	Sensation of location, position and change of body.<br>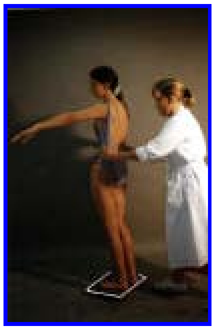 Proprioception exercise on Freeman plate
Proximal	Nearer or closer to a point of reference; opposite of distal.<br>A structure that is located closer to the attached end of a limb.
Psychological Well-being (aim)	Psychological concept including six components: autonomy, environmental mastery, personal growth, positive relationships, purpose in life and self-acceptance (Ryff & Singer, 1998).
Push (brace)	The act of applying force in order to move something away.

Q	
Quality of Life (aim)	Personal satisfaction (or dissatisfaction) with the cultural or intellectual conditions under which you live.

R	
Radiographic Shoulder Height	Radiographic shoulder height (RSH)is determined from the standing anteroposterior radiograph<br>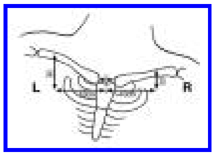<br>The difference betweena.&b.in millimeters represents the RSH
Raimondi Rotation (evaluation)	One of the method to measure vertebral rotation with the Raimondi rule. (see also Nash & Moe, Drerup, Perdriolle, Raimondi)
Range of Motion, R.O.M.	Term used to measure the amount of flexion,extension, and rotation in a joint/extremity.
Rehabilitation Team	Group of allied health care professionals that frequently includes physician, surgeon, orthotist/prosthetists, physical and occupational therapist, social worker and counselor who serve the needs of a patient.
Rehabilitation Treatment (rehab)	The process of restoration of skills by a person who has had an illness or injury so as to regain maximum self-sufficiency and function in a normal or as near normal manner as possible.
Relaxed Posture (rehab)	The position of the human body in the space when the subject do not try to control it actively. (without strain or anxiety)

Relief Area	When fabricating an orthosis or prosthesis, reliefs are made to provide space over a wound or bony prominence.
Respiratory Capacity (rehab)	Concerning the overall function of the pulmonary system. *(see also Vital capacity & Total lung capacity)*
Respiratory Education (rehab)	Process of imparting or acquiring general knowledge, to develop the ability of the respiratory system to take air into the body and the efficiency with which the lungs supply.
Restoration of Sagittal Profile (brace, rehab)	Correction of vicious attitudes or deviations in the sagittal plane to obtain physiological curvatures.<br>Recovery of an harmonic ratio between the thoracic kyphosis and the lumbar lordosis.
Rib hump	Scoliotic convexity, protruding rotated aspect of rib cage.<br>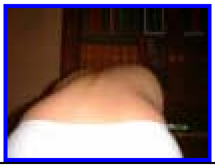
Rib Rotation Prominence (evaluation)	The prominence of the ribs best exhibited on forward bending. *(see also ATI)*<br>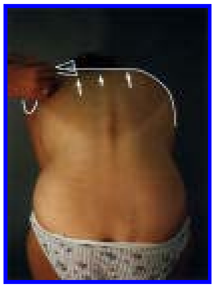 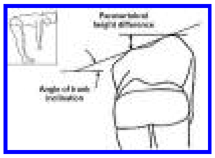
Rib Vertebral Angle	*(see Mehta Rib Vertebral Angle)*
Rigid Brace	
Risser Sign (evaluation)	Used to evaluate skeletal and spinal maturity, this refers to the appearance of a crescent-shaped line of bone formation which appears across the top of each side of the pelvis on plain x-ray.<br>Evaluation of the ossification of the Iliac Crest. It's correlated with vertebral growth stages. It's based on a 6 point scale from 0 (completely immature) to 5 (end of growth):<br>Risser 1: 25% iliac apophysis ossification Anterior Superior iliac spine (anterolateral) Seen in prepuberty or early puberty<br>Risser 2: 50% iliac apophysis ossification Ossification extends halfway across iliac wing Seen immediately before or during growth spurt<br>Risser 3: 75% iliac apophysis ossification. Indicates slowing of growth<br>Risser 4: 100% ossification, with no fusion to iliac crest. Indicates slowing of growth<br>Risser 5: Iliac apophysis fuses to iliac crest. Indicates cessation of growth<br>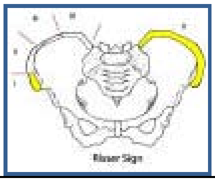
Rotation	Is a rigid body movement which keeps a point fixed; unlike a translation. This definition is applicable both for rotations in a plane (two dimensions) and in space (three dimensions).

Round Back (diagnosis)	Popular term for hyperkyphosis (like hunchback). Some degree of rounded curvature of the spine is normal.

S	
Sacral Inclination	The angle between the line along the posterior border of S1 and the vertical lateral edge of a lateral standing radiograph (or between perpendiculars to these lines).
Sacral Obliquity	An angular deviation of the sacrum from the line drawn parallel to a line across the femoral heads on a supine AP view of the sacrum.
Sacral Slope (evaluation)	Inclination or tilt in a sagittal plane of the first sacral vertebra which diverge from horizontal. (see also lumbosacral angle)<br>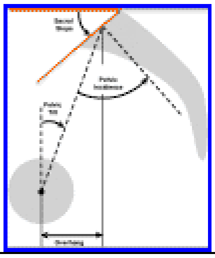
Sacral Spine - (Sacrum)	The curved triangular bone at the base of the spine, consisting of five fused segments of the lower spine that have four foramen on each side. The sacrum articulates (connects) with the last lumbar vertebra and laterally with the pelvic bones.
Sacroiliac Orthosis	Lumbo-Sacral-Pelvic Orthosis.
Sacroiliac Joint	The joint between the ilium and sacrum one each side of the pelvis which has a small amount of motion. It may be a source of low back pain. The ligamentous attachments may become injured as well.
Sagittal Plane	reference plane as seen from the side (as opposed to frontal, horizontal.<br>Pertaining to the side of.. Observes flexion and extension.
Sagittal Plane Restoration (brace, rehab)	Correction of vicious attitudes or deviations in the sagittal plane to obtain physiological curvatures.<br>Recovery of an harmonic ratio between the thoracic kyphosis and the lumbar lordosis. (see Restoration of sagittal profile)
Sagittal Plumbline (evaluation)	Measure of the arrows for the sagittal plane: the difference of distance from the plumbline measured at C7, D12, L3 and S1 with respect to the apex of the thoracic kyphosis.<br>A line usually drawn from C7 from which a weight is suspended to determine verticality in the sagittal plane.<br>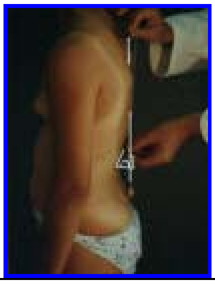
Sagittal Spinal Balance	The vertical alignment of the mid point of C7 with the mid-point of the sacrum in the coronal plane (equates with the term coronal balance) i.e. horizontal distance of the C-7 mid point from the central sacral line.<br>The ratio between the kyphosis and the lordosis measured

	clinically or radiographycally.<br>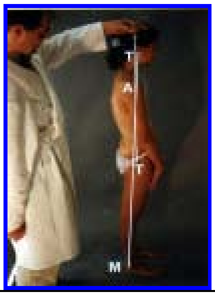
Sagittal T1 Tilt Angle	The ant/post tilt of the T1 vertebral body seen in the sagittal plane on the lateral radiograph. *(see also Coronal T1 Tilt Angle)*<br>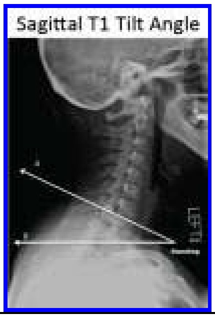
Sagittal Vertical Axis (SVA)	The deviation of the plumbline from the odontoid process in the sagittal plane anterior or posterior to the posterior edge of the top of the sacrum, measured in millimeters.<br>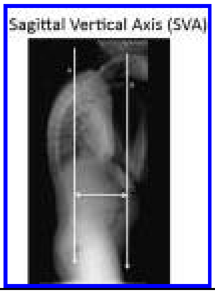
Scheuermann	Holger Werfel Scheuermann (1877-1960) a Danish surgeon serving in Copenhagen published this report in 1921, during the period before the osteochondroses - then know as osteochondritis - were considered similar pathological entities. It appeared near the end of an era when orthopedic disease or surgical eponyms were a mark of distinction. "Scheuermann's disease" entered the list immediately.<br>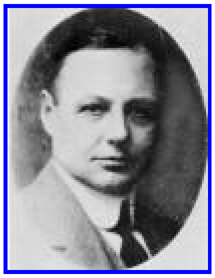 Original paper
Scoliologic ® Cheneau Light Brace	The ScoliOlogiC® off the shelf bracing system enables the CPO to construct a light brace for scoliosis correction from a variety of pattern specific shells to be connected to an anterior and a posterior upright. This brace is called Chêneau light™ brace.

	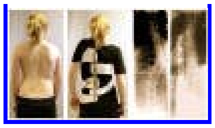 original paper
Scoliometer	A proprietary name for an inclinometer used in measuring trunk rotation.
Scoliosis	Lateral deviation of the normal vertical line of the spine which, when measured by x-ray, is greater than ten degrees. Scoliosis consists of a lateral curvature of the spine with rotation of the vertebrae within the curve. Rotation of the vertebrae also occurs which produces the rib cage and flank muscle asymmetry.
Secondary Scoliosis	Secondary scoliosis in a vertebral pathology such as an osteoid osteoma. It is a painful scoliosis with a strong limitation of the forward bending of the trunk. 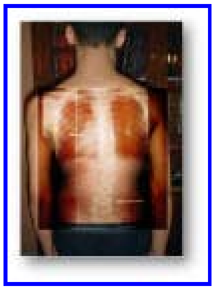 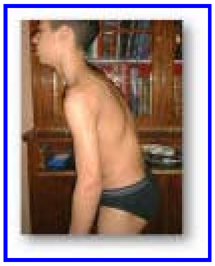 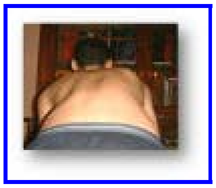 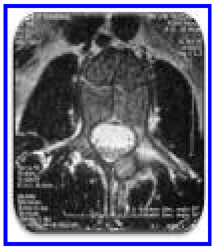
Segmental Rib Vertebra Angle (evaluation)	Usually the Rib Vertebra Angle is calculated from the apical horizontal vertebra where the asymmetry of the posterior part of the rib is the most important. It can be calculated on an upper or lower vertebra.
Sensory Motor Integration (rehab)	Sensory Integration is also known as Sensory Motor Integration, a global understanding of how the human body and mind interact with the environment and operate on a daily basis. Sensory Integration theory teaches that the ability of the vestibular system to modulate sensory input has a powerful impact on the development of functional skills.
Shift (brace)	A change in place, position, or frequency.
Side-shift (rehab)	Exercise for thoraco-lumbar scoliosis described by Min Mehta. The child moves with a translation of the trunk to the convexity of the curve while keeping fixed both scapular and pelvic girdles. 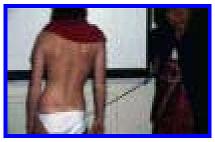 Min Mehta demonstrating the side-shift (London – doc de Mauroy 1982)
Sitting Height (evaluation)	A measure of the vertical distance (taken along the back) from the table surface to the crest of the head as the subject sits erectly on the table.
Skeletal age	The age obtained by comparing PA x-ray of the left wrist and hand with the standards of the Greulich and Pyle atlas.
Spaces (brace)	A particular area or cavity within the orthosis – non contact area.
Spina Bifida	Paralysis caused by congenital defect of the spinal column.
Spinal Balance	Spinal balance is cumulative. Unless all the translational and angular displacements of vertebrae in one direction are countered by opposite displacements and angulations of equal magnitude, the spine is unbalanced.
Spondylolisthesis	An anterior displacement (slipping) of a vertebra on the

	adjacent lower vertebra. There are several causes for this. There can be varying degrees of displacement so the vertebra and the spine above that vertebra are displaced forward in relationship to the vertebrae below. It is frequently due to a developmental defect or the result of a fracture.<br><br>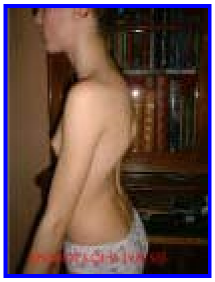 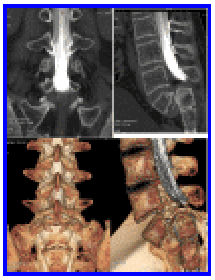 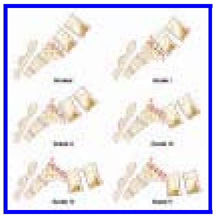 Meyerding's grading
Spondylolysis	(also referred to as a stress fracture or a pars fracture) - Fracture of a posterior portion of the vertebra with a defect in the neural arch between the superior and inferior facets of vertebrae without separation at the defect and therefore no displacement of the vertebrae. It may be unilateral or bilateral and is sometimes due to a developmental defect but may be secondary to an traumatic fracture.
Spontaneous Posture (rehab)	neutral, every-day posture, not totally relaxed<br><br>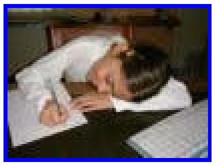 Spontaneous position to write at a desk
Stabilisation Exercises (rehab)	Type of therapeutic exercise that focuses on enhancing and improving strength, endurance.
Stabilisation of the curve (brace, rehab)	To make stable – stop the curve from progressing.
Standard Posture	Ideal alignment is a balanced posture in which positioning is centered and relaxed for all the joints of the body.
Stationary Intensive Rehabilitation (SIR)	A special kinesitherapeutic method used on an in-patient basis (hospital department, sanatorium or a similar form of health care).
Stiffness (evaluation)	
Stop (brace)	(translation of the french “butée”) Part of the brace theoretically without contact with the body to avoid the creation of a scoliotic counter curve.
Structural Scoliosis Curvature (organic)	A measured spinal curve in the coronal plane in which the Cobb measurement fails to correct past zero on supine maximal voluntary lateral side bending x-ray.
Supine	Lying on your back.
Surface Topography (evaluation)	Study of the three dimensional shape of the surface of the back.<br><br>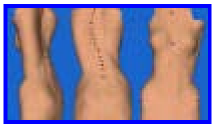 Full 3d instantaneous Orten imaging

T

Team (brace, rehab)	Members of the allied health care group that work together to provide comprehensive quality care to the patients and families under their service.
Technician-Orthotic	Person trained to fabricate, repair and maintain orthoses under the supervision of an orthotist.
Thoracic extension, thoracic pad	Refers to the module plastic and pad extending cephalad from the iliac crest roll and intended to contact lower thoracic ribs.
Thoracic (Dorsal) Spine	Twelve spinal segments (T1-T12) incorporating the 12 ribs of the thorax. Other than a slight increase in size from top to bottom, they are fairly uniform in appearance.
Thoracic Scoliosis Curvature	Any spinal curvature in which the apex of the curve is between the second and eleventh thoracic vertebrae. 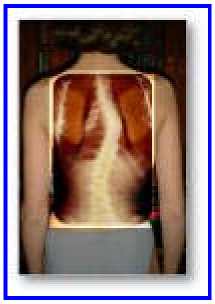 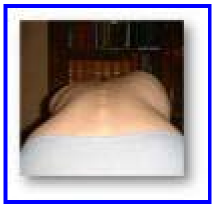 Thoracic scoliosis
Thoracic Ratios	Chest radiograph to show the method of measurement for calculation of thoracic ratios.<br>D = distance measured from midpoint of the distal end-plate of each vertebral body (TI-12) to the outline of the lateral border of the right - DR(left DL) thoracic cage.<br>H = distance from TI-12<br>On each chest radiograph, the outline of the lateral border of the thorax is drawn<br>(Fig). Next, the midpoint of the distal end-plate at each vertebral body from T1-12 is marked. Then, at each segment, the distance from the middle of the end-plate to the outline of each of the right and left thoracic cage is measured. These distances are standardised by dividing by the measured Tl-12 distance. They are termed segmental right and left thoracic ratios (TRs). Ratios are also calculated segmentally for the total width of the chest (right plus left measured lengths). 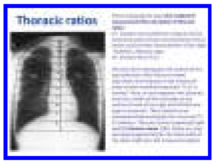 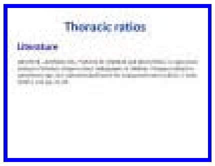
Thoracolumbar Scoliosis Curvature	Any spinal curvature that has its apex at the twelfth thoracic or first lumbar vertebra.

	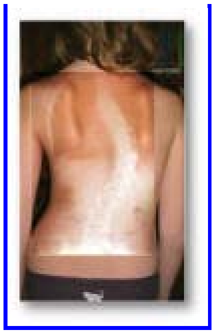 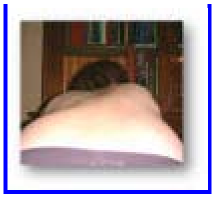 Thoracolumbar Scoliosis
Thoracolumbosacral Orthosis (TLSO)	A type of brace immobilizing the thoracic lumbar and sacral spine. This may be used to help stabilize/ prevent progression of scoliosis curve(s) while a child is growing, or to immobilize the spine after surgery.
Thrust (brace)	To push or drive with force in an orthotic device.
Tolerability (brace)	It's a key principle of bracing, since it allows compliance, so increasing efficacy.
Torsion	Torsion (mechanics) In solid mechanics, torsion occurs when an object is twisted or screwed around its axis. Torsion can be the result of an applied torque. It is a kind of shear stress. For circular sections, the shearing stress at a point on a transverse plane is always perpendicular to the radius to the point.<br>Torsion has two meanings: it is a property both of the vertebral body line (geometric torsion) and of the vertebrae themselves (mechanical torsion).<br>Mechanical torsion is the relative rotation and/or vertebral deformations between lines joining similar landmarks on vertebrae. It is unclear to what extent mechanical torsion results from relative motion between vertebrae, and to what extent it involves distortion of the vertebrae themselves.
Total lung capacity (TLC)	The maximum volume to which the lungs can be expanded with the greatest possible inspiratory effort.
TRACE (evaluation)	acronym of Trunk Aesthetic Clinical Evaluation, is a 12 points (0-11) scale for the clinical evaluation of the aesthetic appearance of the trunk. TRACE is based on four sub-scales: shoulders, scapulae and waist and the hemi-thorax. The final score is given by the sum of the single item plus 1.<br>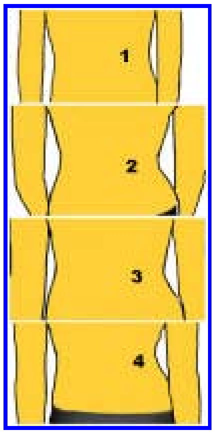 TRACE for Waist triangle asymetry
Transitional Point (evaluation)	A point at which the spinal curve change of orientation. At this point we have a transitional vertebra.
Transverse Plane (evaluation)	(Also called the horizontal plane, axial plane, or transaxial plane) is an imaginary plane that divides the body into superior and inferior parts. It is perpendicular to the coronal and sagittal planes.

Trimline	The outline of the brace. Usually high-grade HyperFoam Trimline wraps provide comfort and durability.
Trochanter extension, trochanter pad	Commonly, plastic is left extending caudad to cover one greater trochanter. When needed, a pad is also used on the inner surface of the extension. The trochanter extension is essential to provide balance for the brace and avoid sideward tilting relative to the pelvis. Generally, the trochanter extension is placed on the side toward which L5 tilts.
Truncal Balance	In a translational sense it involves the mass or volume of displaced segments, as well as the magnitude of their displacements. In keeping with this report defining terminology only for the spine, no attempt is made to define truncal balance.<br>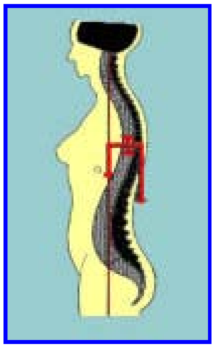 Balance in a sagittal plane
Trunk Shift	Trunk shift (TS)in the coronal plane is determined by measuring the perpendicular distance from the center sacral line to a line that bisected the distance from the lateral edges of the rib margins in the mid thoracic area. *(Keithet al Spine 2008)*<br>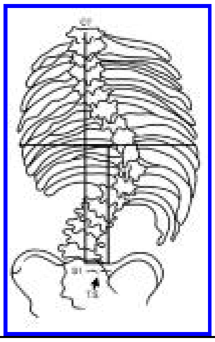

# U	
Upright	Vertical side bar used in an orthosis.

# V	
Vector Force (brace)	A particular type of vector representing a force operating in a defined direction and magnitude.
Vertebra	One of the 33 bones of the spinal column. A cervical, thoracic, or lumbar vertebra has a cylindrically shaped body anteriorly and a neural arch posteriorly (composed primarily of the laminae and pedicles as well as the other structures in the posterior aspect of the vertebra) that protect the spinal cord. The plural of vertebra is vertebrae.

	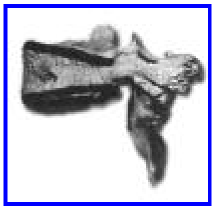 (doc Perdriolle)
Vertebra Centroid	Concept: The mid point of a vertebral body
Vertebral Body Line	Concept: The curved line that passes through the vertebra centroids. Geometric principles, relying on vector algebra (Kreyszig, 1979, pp 367-382) can be used to define characteristics of a line in space, and can therefore be applied to the vertebral body line. 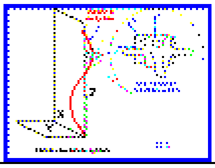
Vertebral Column	The flexible supporting column of vertebrae separated by discs and bound together by ligaments.
Vertebral Tilt	Vertebral angulation to the horizontal in the coronal plane, measured from specified landmarks on the vertebra in a standing radiograph (normally lines drawn on the upper or lower endplate).
Vertebral Wedge Angle	*see also Disc Wedge Angle*
Vicious Circle	Hypothesis that in progressive scoliosis vertebral body wedging during adolescent growth results from asymmetric muscular loading in a "vicious cycle". 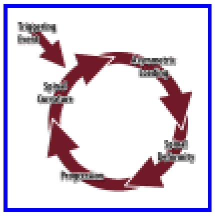 Ian stokes' vicious circle
Visibility (brace)	Another principle of bracing implies to reduce as much as possible visiblity to increase compliance.
Vital Capacity	The amount of air that can be forcibly expelled from the lungs after breathing in as deeply as possible.

W	
Wearability (brace)	Term used to describe the extent to which an orthosis is able to be tolerated while being worn by a patient.<br>A principle of correct bracing.
Window (brace)	An opening in an orthosis to allow space or the ability to view the internal aspect of the orthosis. (this one needs work)

**Acronyms & Initialisms**	**Definition**
3C	Functional 3 curve pattern according to Lehnert-Schroth
4C	Functional 4 curve pattern according to Lehnert-Schroth

ADL	Activities of Daily Living
AIS	Adolescent Idiopathic Scoliosis
AP	Antero-Posterior (front plane)
AS	Adult Scoliosis
ASC	Active Self Correction
ASIS	Antero Superior Iliac Spine
ATI	Angle of Trunk Inclination
ATR	Angle of Trunk Rotation
BSR	Back Surface Rotation
CADCAM	Computer Aided Design, Computer Aided Manufacture.
CCT	Clinical Controlled Trial
COM	Center of Mass
COP	Center of Pressure
CPO	Certified Prosthetist Orthotist
CS	Congenital Scoliosis
CTAS	Crude Trunk Asymmetry Score
CTLSO	Cervico-Thoraco-Lumbar-Sacral-Orthesis
CVA	Coronal Vertical Axis
DAPI	Deformity in the Axial Plane Index
DRSC	Double Rib Contour Sign
FTB	Full Time Bracing
HS	Hump Severity
IS	Idiopathic Scoliosis
ISIS	Integrated Shape Imaging System
NTB	Night Time Bracing
OBS	Overall Balance Summation
POTSI	Posterior trunk symmetry index
PSIS	Postero Siperior Iliac Spine
PGS	Pubertal Growth Spurt
PT	Physical Therapist.
QoL	Quality of Life
RCT	Randomised Control Trial
RSC	Rigo System Cheneau
SEM	Standard Error of Measurement
SPORT	Symmetric Patient-Oriented Rigid Three-dimensional active
TLSO	Thoraco-Lombo-sacral-Orthesis
TRACE	Trunk Aesthetic Clinical Evaluation
SEAS	Scientific Exercises Approach to Scoliosis
SRS	Scoliosis Research Society
SSSP	School Scoliosis Screening Program
ST	Surface Topography
SVA	Sagittal Vertical Axis
VA	Volumetric Asymmetry

QUOTATIONS

Life is short, art is long; opportunity is fleeting, experiment is fallible; judgment is difficult.
Hippocrate

"The treatment of a confirmed scoliosis is a perpetual and unpleasant lesson in humility"
Roederer

"Among all those who did not want to subject themselves to control of the facts of their pathogenic assertions, and among all those who did not want to examine closely the relevant criticisms of the results of the treatments about which they speak enthusiastically without bringing of proof, indeed, few saw their assertions stand the test of time."
Pierre Stagnara

"Without the theory, the practice becomes only a boring habit. Only the theory allows us to forge ahead and to develop the spirit of invention."
Louis Pasteur

" Science without conscience is only destruction of the soul"
François Rabelais

- It is easy to encourage the research; it is more difficult to manage its contradictions.
- Scoliosis is a disease which lasts all of a person's life. The patient with scoliosis is a low back pain patient in power.
- Scoliosis is a serious illness which has social, professional, and psychological consequences.
- Experience is not always transferable, but the reflection by some people always opens roads for the others.
- In the spine, "the soft" is probably more important than "the hard."
- The radiograph shows only the "Chinese shadows" of the reality.
- The computer returns only what we gave it to eat. The computer cannot and never should supplant the observation and the clinical assessment of the clinician.
- The spirit of invention must be always preceded by the necessity of the knowledge.

Jean Dubousset

Written by: Theodoros B Grivas, Fabio Zaina, Jean Claude de Mauroy, Stefano Négrini, Paolo Pizzetti, Patrick Knott

This site complies with the HONcode standard for trustworthy health information:
verify here.

Last update: June 3, 2010
